# Superoxide dismutase SOD-1 modulates *C. elegans* pathogen avoidance behavior

**DOI:** 10.1038/srep45128

**Published:** 2017-03-21

**Authors:** Alexander M. Horspool, Howard C. Chang

**Affiliations:** 1 Department of Biological Sciences, Binghamton University, SUNY, Binghamton, NY 13902, USA

## Abstract

The *C. elegans* nervous system mediates protective physiological and behavioral responses amid infection. However, it remains largely unknown how the nervous system responds to reactive oxygen species (ROS) activated by pathogenic microbes during infection. Here, we show superoxide dismutase-1 (SOD-1), an enzyme that converts superoxide into less toxic hydrogen peroxide and oxygen, functions in the gustatory neuron ASER to mediate *C. elegans* pathogen avoidance response. When *C. elegans* first encounters pathogenic bacteria *P. aeruginosa*, SOD-1 is induced in the ASER neuron. After prolonged *P. aeruginosa* exposure, ASER-specific SOD-1 expression is diminished. In turn, *C. elegan*s starts to vacate the pathogenic bacteria lawn. Genetic knockdown experiments reveal that pathogen-induced ROS activate *sod*-*1* dependent behavioral response non cell-autonomously. We postulate that the delayed aversive response to detrimental microbes may provide survival benefits by allowing *C. elegans* to temporarily utilize food that is tainted with pathogens as an additional energy source. Our data offer a mechanistic insight into how the nervous system mediates food-seeking behavior amid oxidative stress and suggest that the internal state of redox homeostasis could underlie the behavioral response to harmful microbial species.

Food is often tainted with pathogens^1^,^2^. Ingestion of pathogenic microbes triggers reactive oxygen species (ROS) production in the intestine^3^,^4^,^5^,^6^. Although ROS can serve as signaling molecules to activate protective signaling pathways^6^,^7^,^8^,^9^,^10^,^11^, elevation of ROS (e.g., •O2−, •OH) causes oxidative stress. The targets of free-radical mediated damage are proteins, lipids and DNA^10^,^11^. Therefore, the failure to maintain cellular and systemic ROS concentration results in physiological decline, as oxidized proteins threaten cellular physiology.

Maintaining physiological homeostasis is essential to survival. In *C. elegans*, the nervous system plays a major role in modulating stress response. For example, the thermal sensory neuron AFD senses heat stress and activates heat shock response at distal tissue via serotonin signaling^12^. The octopamine receptor OCTR-1 acts in the ASH and ASI sensory neurons to downregulate ER unfolded protein response. In turn, it further suppresses the innate immune signaling activation in the intestine^13^. TRX-1, a thioredoxin, promotes nuclear localization of intestinal SKN-1 in a cell non-autonomous fashion from distal ASJ A neurons^14^. These mechanisms demonstrate the importance of the nervous system in detecting adverse environmental cues and maintaining physiological homeostasis.

Microbes in the digestive tract also modulate host metabolic activity and physiology^15^. For example, mammalian gut indigenous bacteria increase the secretion of peripheral serotonin and subsequently enhance gastrointestinal movement^16^. Although *C. elegans* can grow axenically on defined culturing ingredients without bacteria as its food source, recent studies show that bacterial diet plays an important role in aging and intestine health^17^,^18^,^19^,^20^. Microbiota (the community of microbial species) in the intestine can be commensal or pathogenic^21^. Prior to entering the intestine of *C. elegans*, bacteria are normally broken down in the pharynx. However, several human opportunistic pathogens including *Pseudomonas aeruginosa, Serratia marcescens* and *Salmonella typhimurium* have been found to colonize the *C. elegans* intestine^17^,^22^,^23^,^24^. Therefore, the *C. elegans* host and pathogen model offers a unique tool to study pathogenic microbiota and their role in affecting host physiology^25^,^26^,^27^.

When *C. elegans* is transferred to a petri dish that contains *P. aeruginosa* PA14, *C. elegans* will initially remain inside the bacterial lawn and feed on *P. aeruginosa*. Ingestion allows the accumulation of *P. aeruginosa* in the intestine^17^, which in turn triggers ROS production^4^,^5^. After prolonged exposure to *P. aeruginosa, C. elegans* will vacate the *P. aeruginosa* lawn^24^,^28^. Several sensory modalities including olfactory, chemosensory, and mechanosensory have been shown to mediate *C. elegans* behavioral response to pathogens^28^,^29^,^30^. In addition, it has been demonstrated that neuropeptide receptor NPR-1, which encodes *C. elegans* homolog of mammalian neuropeptide Y receptor, is one of the behavioral determinants of pathogen susceptibility^24^,^28^,^31^. However, how ROS mediate *C. elegans* physiological and behavioral responses to pathogens remains elusive.

Superoxide dismutase-1 (SOD-1) is an antioxidant enzyme. SOD-1 catalyzes the dismutation of superoxide into less toxic hydrogen peroxide and oxygen. Mutations in *sod*-*1* are linked to a familial form of amyotrophic lateral sclerosis (ALS), a progressive neurodegenerative disease that affects nerve cells^32^,^33^,^34^. Although the pathological mechanism of ALS is still under debate, one of the prevailing hypotheses suggests that the degeneration of nerve cells is caused by chronic oxidative stress in the nervous system^35^,^36^,^37^.

A recent study in budding yeast highlights a dual role of SOD-1 in maintaining respiratory homeostasis and glucose metabolism^38^. In growth media with high glucose concentration, SOD-1 suppresses the degradation of casein kinase 1, a signaling molecule that represses respiratory metabolism. In *C. elegans*, SOD-1 is elevated by environmental ROS challenge. Lack of SOD-1 activity causes life span reduction^39^. Here, we postulate that SOD-1 may act as a sensor of redox homeostasis and mediate behavioral response to food. As a result, SOD-1 may alleviate oxidative stress triggered by foodborne agents such as pathogenic microbes.

In this study, we examined how SOD-1 mediates pathogen avoidance behavior in *C. elegans*. When *C. elegans* first encounters *P. aeruginosa*, SOD-1 is elevated in the gustatory neuron ASER. The elevation of SOD-1 eventually returns to baseline level after prolonged exposure to *P. aeruginosa*. In turn, *C. elegans* starts to avoid *P. aeruginosa*. We also found that SOD-1 is localized at the sensory cilium of ASER. By introducing a mutation that disrupts the structure of sensory cilium and by performing RNA interference to a gene that enhances ROS production, we were able to block SOD-1 induction in the ASER neuron. The lack of SOD-1 and the ablation of ASER neuron exacerbate *P. aeruginosa* aversion. This suggests that SOD-1 functions in the ASER neuron to inhibit the aversive response to *P. aeruginosa*. Together, our findings demonstrate that SOD-1 modulates pathogen avoidance behavior by integrating the external sensory stimuli and the internal state of redox reaction. In the arms race between host and pathogen, the delayed aversive response to food that contains small quantities of pathogens may allow the host to activate protective responses and prepare for possible infection.

## Results

### *sod*-*1* mutant animals elicit a heightened *P. aeruginosa* lawn avoidance phenotype

**Figure 1:**
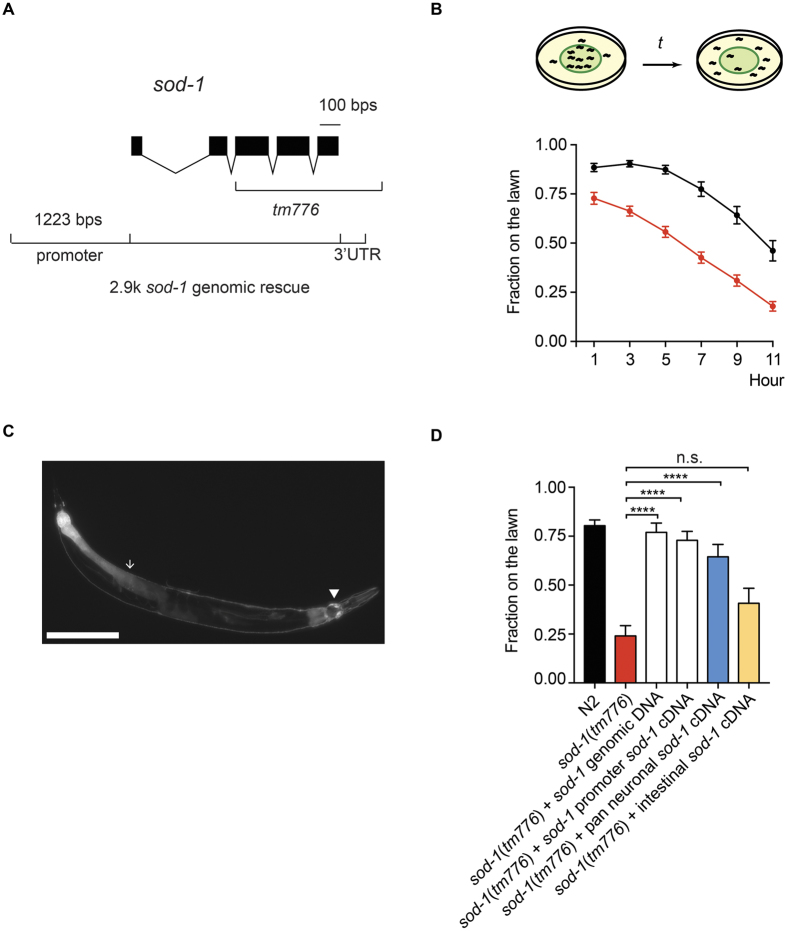
SOD-1 modulates *P. aeruginosa* avoidance behavior. (**A**) The 2.9 kb region of *sod*-*1* genomic fragment. The deleted region of the *sod*-*1(tm776*) mutant is indicated. (**B**) Top: schematic of *P. aeruginosa* avoidance assay. *C. elegans* was transferred to plates containing a lawn of *P. aeruginosa*. Lawn occupancy was scored over time. Bottom: time course of the *P. aeruginosa* lawn occupancy. Black indicates wild-type N2 strain; red indicates *sod*-*1(tm776*) mutant strain. *N* = 20. Error bars represent standard error of the mean. (**C**) Fluorescence micrograph of *bosEx1*[*sod*-*1p*::GFP]. GFP fluorescence is found in the nervous system (triangle) and in the intestine (arrow). Scale bar indicates 50 μm. (**D**) *P. aeruginosa* lawn occupancy of *C. elegans* strains assayed at t = 7 h. *sod*-*1* genomic rescue contains the 2.9 kb *sod*-*1* genomic fragment indicated in (**A**). *sod*-*1* cDNA rescue contains *sod*-*1* cDNA driven by the *sod*-*1* promoter. Pan-neuronal and intestinal rescue constructs are *sod*-*1* cDNA under control of the *unc*-*119* promoter and the *ges*-*1* promoter. ****Represents *p* < 0.0001, n.s. not significant, as determined by one-way ANOVA, followed by Bonferroni’s multiple comparison test. *N* = 12. Error bars represent standard error of the mean.

To explore if SOD-1 mediates behavioral response to pathogens, we first exposed *sod*-*1(tm776*) mutant to a lawn of *P. aeruginosa* and observed its behavior over time (Fig. 1A and B). Based on the sequencing results, *sod*-*1(tm776*) contains a lesion that deletes the majority of the *sod*-*1* coding region and the 3′UTR (Fig. 1A). Therefore, we hypothesized that *sod*-*1(tm776*) may act as a null mutation. We found that *sod*-*1(tm776*) mutant animals leave the *P. aeruginosa* lawn earlier than wild type N2 control. For example, after 7 hour exposure to *P. aeruginosa*, only 42% of the *sod*-*1(tm776*) animals remained inside of *P. aeruginosa* lawn compared to 77% lawn occupancy of the wild type animals (Fig. 1B). We then generated *sod*-*1* rescue constructs by introducing a 2.9 kb *sod*-*1* genomic fragment and by expressing *sod*-*1* cDNA under control of the *sod*-*1* promoter (Fig. 1A). We found that both *sod*-*1* genomic and *sod*-*1* cDNA constructs rescued the *sod*-*1(tm776*) pathogen avoidance phenotype to the wild type level. At 7 hour, 76% of *sod*-*1* genomic rescue and 72% of *sod*-*1* cDNA rescue animals remained on the *P. aeruginosa* lawn (Fig. 1D). Thus, we concluded that SOD-1 represses the avoidance response to *P. aeruginosa*.

### SOD-1 functions in the nervous system to modulate the behavioral response to pathogen

We investigated the expression pattern of SOD-1 by generating a strain that carries the *sod*-*1p*::GFP transgene. We observed GFP signals in the intestine. We also observed GFP signals in the head and tail neurons. GFP fluorescence signals were also located along the ventral and dorsal nerve cords (Fig. 1C). Therefore, SOD-1 is present in the nervous system and in the intestine.

To investigate the tissue-specific requirement of *sod*-*1* in *C. elegans*, we performed tissue-specific *sod*-*1* rescue experiments using pan-neuronal (*unc*-*119*) and intestinal (*ges*-*1*) promoters^40^,^41^. We found that pan-neuronal expression of *sod*-*1* strongly rescues the pathogen avoidance phenotype of *sod*-*1(tm776*) mutant. By contrast, intestinal expression of *sod*-*1* did not rescue the *sod*-*1(tm776*) phenotype (Fig. 1D). These results demonstrate that SOD-1 functions in the nervous system to regulate the behavioral response to *P. aeruginosa*.

### SOD-1 functions in the gustatory neuron ASER to delay the avoidance response to *P. aeruginosa*

**Figure 2:**
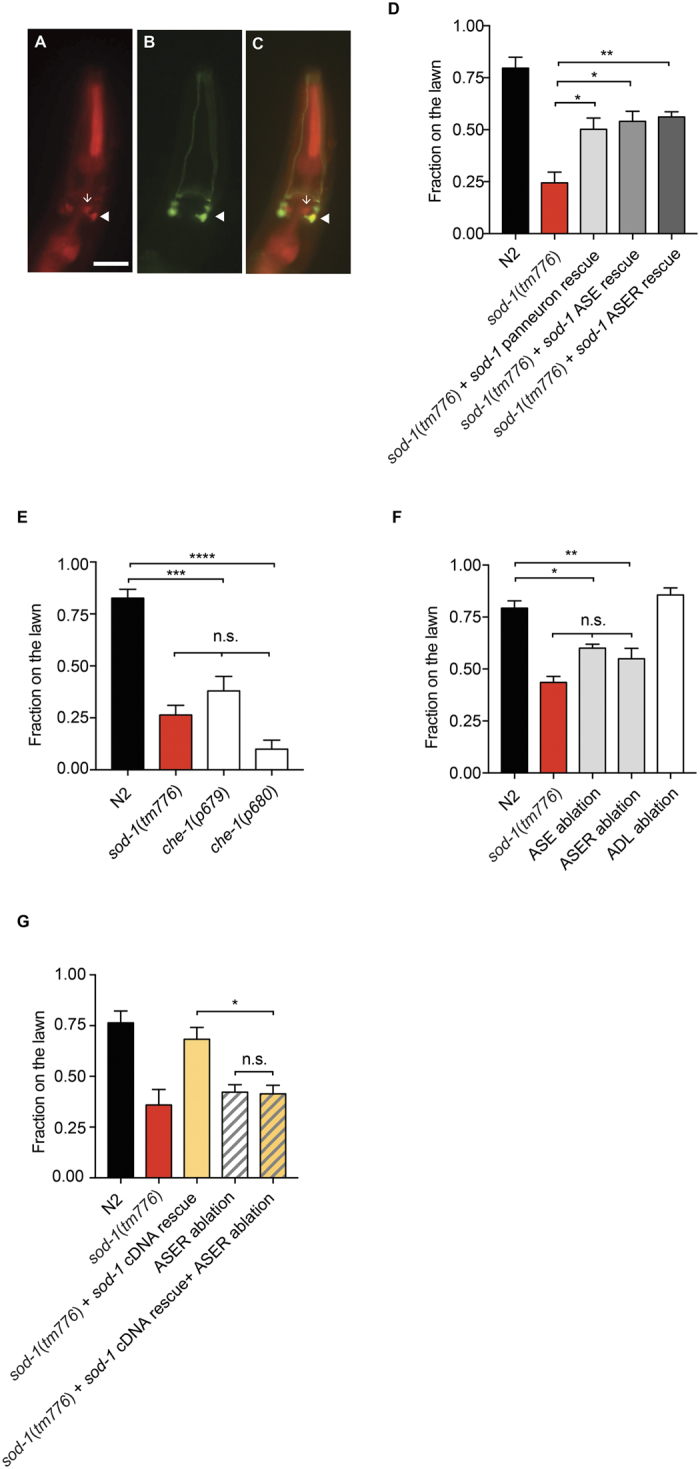
SOD-1 functions in the ASER neuron to delay *P. aeruginosa* avoidance response. (**A**–**C**) Colocalization of *sod*-*1p*::RFP and *flp*-*6p*::GFP reporters. (**A**) Fluorescence micrograph of *sod*-*1p*::RFP. (**B**) Fluorescence micrograph of the ASE neuron pair marker *flp*-*6p*::GFP. (**C**) Merge of (**A**) and (**B**). Triangle represents ASER. Arrow represents ADLR. Scale bar indicates 10 μm. (**D**–**G**) *P. aeruginosa* lawn occupancy of *C. elegans* strains assayed at t = 7 h. *N* = 9–18. Error bars represent standard error of the mean. (**D**) *sod*-*1* ASE rescue construct is *flp*-*6p*::*sod*-*1* cDNA. *sod*-*1* ASER rescue construct is *gcy*-*5p*::*sod*-*1* cDNA. **Represents *p* < 0.01, *represents *p* < 0.05, as determined by one-way ANOVA, followed by Tukey’s multiple comparison test. (**E**) *che*-*1* mutants elicit heightened *P. aeruginosa* avoidance. ****Represents *p* < 0.0001, ***represents *p* < 0.001, n.s. not significant, as determined by one-way ANOVA, followed by Tukey’s multiple comparison test. (**F**) ASE, ASER and ADL ablation are transgenic animals that carry an extra-chromosomal array of *flp*-*6p*::*csp*-*1b, gcy*-*5p*::*csp*-*1b* or *sre*-*1p*::*csp*-*1b*, respectively. **Represents *p* < 0.01, *represents *p* < 0.05, n.s. not significant, as determined by one-way ANOVA, followed by Tukey’s multiple comparison test. (**G**) ASER ablation abolishes the delayed *P. aeruginosa* avoidance phenotype contributed by SOD-1 cDNA rescue in *sod*-*1(tm776*) background. ASER ablation contains integrated *gcy*-*5p*::*csp*-*1b. sod*-*1* cDNA rescue contains integrated *bosIs2* [*sod*-*1p*::*sod*-*1* cDNA::GFP]. *Represents *p* < 0.05, n.s. represents not significant, as determined by one-way ANOVA, followed by Tukey’s multiple comparison test.

Sensory neurons play a major role in modulating *C. elegans* behavioral response to *P. aeruginosa*^28^,^29^,^30^. We noticed that some of the head neurons where SOD-1 is expressed elicit the morphology that resembles amphid sensory neurons (Fig. 1C). To identify these neurons, we generated a *sod*-*1p*::RFP reporter strain and used amphid sensory neuron GFP reporters to perform colocalization experiments. Consistent with results from a previous investigation^39^, we confirmed that SOD-1 is present in the ADL neuron pair (Fig. 2A) and is occasionally present in the ASI and ASK neuron pairs (data not shown). In addition, we discovered consistent RFP signals in an additional amphid sensory neuron. This additional sensory neuron is localized more ventrally and posteriorly to the ASK-ADL-ASI cluster. By performing a colocalization experiment using *flp*-*6p*::GFP (an ASE neuron pair marker), we confirmed the additional sensory neuron is the gustatory neuron ASER (Fig. 2C).

The ASE neuron pair is a pair of bilaterally positioned gustatory neurons that detect water-soluble chemicals^42^. ASE neuron pair contains ciliated dendritic endings, which are exposed to the environment via an opening of the cuticle at the anterior tip, called buccal cavity^43^. The left/right asymmetrical expression of guanylate cyclases in ASEL (left) and ASER (right) neurons allows the ASEL and ASER neurons to discern different tastes^44^,^45^. To determine if SOD-1 is required in the ASE neurons to regulate *C. elegans* pathogen avoidance behavior, we expressed *sod*-*1* cDNA in the ASE neuron pair and in the ASER neuron. Expressing SOD-1 in the ASER neuron rescued the *sod*-*1(tm776*) pathogen avoidance phenotype. ASER-specific *sod*-*1* rescue elicited a similar lawn occupancy phenotype compared to the pan-neuronal rescue of *sod*-*1* (Fig. 2D). Our results confirm that SOD-1 functions in the ASER neuron to modulate behavioral response to *P. aeruginosa*.

In order to determine if the ASE neuron pair plays a role in behavioral response to *P. aeruginosa*, we acquired *che*-*1* mutants and examined their pathogen avoidance phenotype. CHE-1 encodes a C2H2-type zinc-finger transcriptional factor and is required for ASE cell fate determination. *che*-*1* mutant animals elicit no morphological defects in chemosensory neurons. Instead, the ASE neuron pair fails to express ASEL- and ASER-specific guanylate cyclases in the *che*-*1* mutant background^43^,^45^. We observed an enhanced pathogen avoidance phenotype in both *che*-*1(p679*) and *che*-*1(p680*) loss-of-function mutants (Fig. 2E). These results suggest that the ASE neuron pair inhibits behavioral avoidance to *P. aeruginosa*.

To further test if the ASER neuron itself modulates *C. elegans* pathogen avoidance behavior, we performed ablation experiments using CSP-1b caspase to induce apoptosis in the neuron of our interest^28^,^46^. We generated transgenic animals that carry *csp*-*1b* under control of the *flp*-*6* (ASE pair), *gcy*-*5* (ASER), and *sre*-*1* (ADL pair) promoters. We observed an enhanced pathogen avoidance phenotype in animals without the ASER neuron. However, animals without the ADL neuron pair did not elicit enhanced pathogen avoidance behavior (Fig. 2F). We also obtained a transgenic strain that ablates the ASEL neuron^45^. The ablation of ASEL neuron did not enhance the pathogen avoidance phenotype (Supplementary Figure 1). Together, these data suggest that the ASER neuron represses behavioral response to *P. aeruginosa*.

Finally, we introduced integrated *sod*-*1* rescue and ASER ablation transgenes into the *sod*-*1(tm776*) background and observed its *P. aeruginosa* lawn occupancy phenotype. By ablating the ASER neuron, we abolished the delayed pathogen avoidance phenotype contributed by the *sod*-*1* rescue in *sod*-*1(tm776*) background (Fig. 2G). We concluded that SOD-1 functions in the ASER neuron to inhibit the aversive response to *P. aeruginosa*.

### SOD-1 diminishes oxidative stress triggered by *P. aeruginosa*

**Figure 3:**
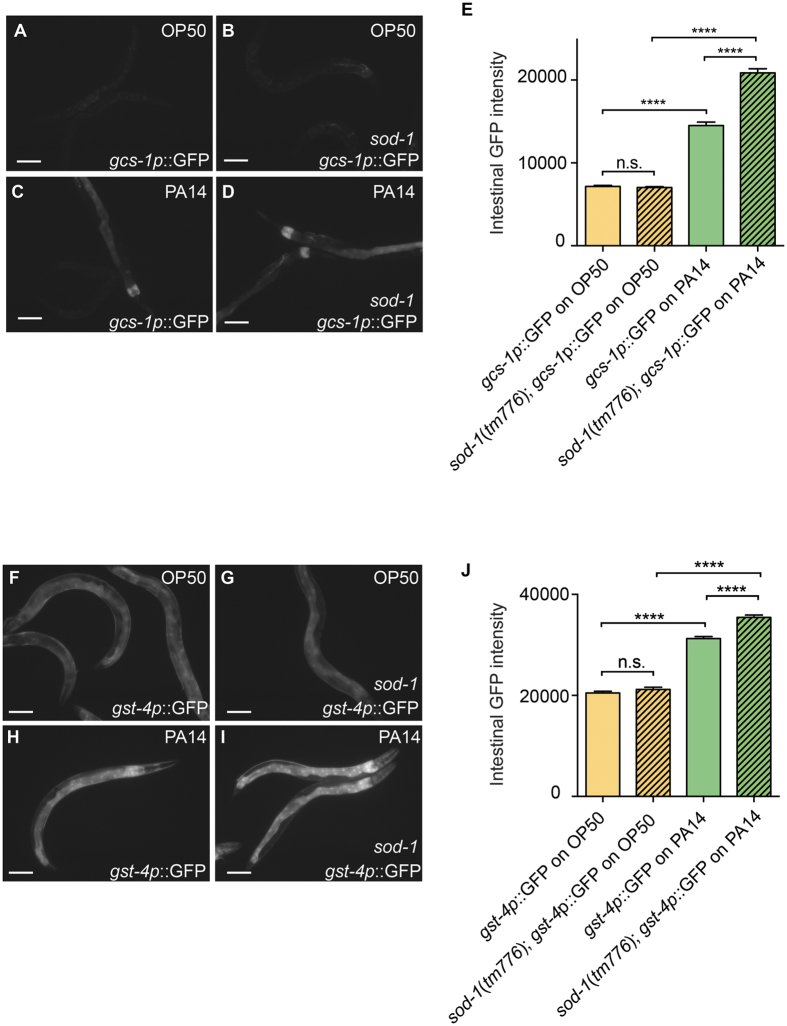
SOD-1 alleviates pathogen-induced oxidative stress in the intestine. (**A**–**D**) Fluorescence micrographs of oxidative stress reporter *gcs*-*1p*::GFP after 2 hour *E. coli*. (OP50) exposure or 2 hour *P. aeruginosa* (PA14) exposure. (**A**,**C**) *gcs*-*1p*::*GFP* in wild type. (**B**,**D**) *gcs*-*1p*::GFP in *sod*-*1 (tm776*) mutant background. Scale bar indicates 50 μm. (**E**) Average fluorescence intensity of *gcs*-*1p*::GFP. ****Represents *p* < 0.0001, n.s. not significant, as determined by student’s *t* test. *N* = 20–25. Error bars represent standard error of the mean. (**F**–**I**) Fluorescence micrographs of oxidative stress reporter *gst*-*4p*::GFP after 2 hour *E. coli*. (OP50) exposure or 2 hour *P. aeruginosa* (PA14) exposure. (**F**,**H**) *gst*-*4p*::GFP in wild type. (**G**,**I**) *gst*-*4p*::GFP in *sod*-*1 (tm776*) mutant background. Scale bar indicates 50 μm. (**J**) Average fluorescence intensity of *gst*-*4p*::GFP. ****Represents *p* < 0.0001, n.s. not significant, as determined by student’s *t* test. *N* = 20–25. Error bars represent standard error of the mean.

Ingestion of *P. aeruginosa* triggers ROS production and activates *gcs*-*1p*::GFP and *gst*-*4p*::GFP reporters in the intestine^4^,^47^. *gcs*-*1* encodes γ-glutamine cysteine synthetase heavy chain and *gst*-*4* encodes glutathione S-transferase. Both GCS-1 and GST-4 are antioxidant enzymes that alleviate oxidative stress^48^,^49^. To examine if SOD-1 alleviates pathogen-induced oxidative stress, we exposed *gcs*-*1p*::GFP, *gst*-*4p*::GFP with or without the *sod*-*1(tm776*) deletion to a lawn of *E. coli* OP50 or to a lawn of *P. aeruginosa* PA14. When animals were fed on *E. coli, gcs*-*1* and *gst*-*4* fluorescence signals were not elevated. In contrast, we observed increased *gcs*-*1* and *gst*-*4* fluorescence signals in the intestine at 2 hour when animals were fed on *P. aeruginosa*. We found that *sod*-*1* mutation further enhances the fluorescence intensity of *gcs*-*1* and *gst*-*4* reporters when exposed to *P. aeruginosa* (Fig. 3). Our results demonstrate that SOD-1 alleviates oxidative stress triggered by *P. aeruginosa*.

### SOD-1 is localized at the sensory cilium and is induced by *P. aeruginosa* in the ASER neuron

**Figure 4:**
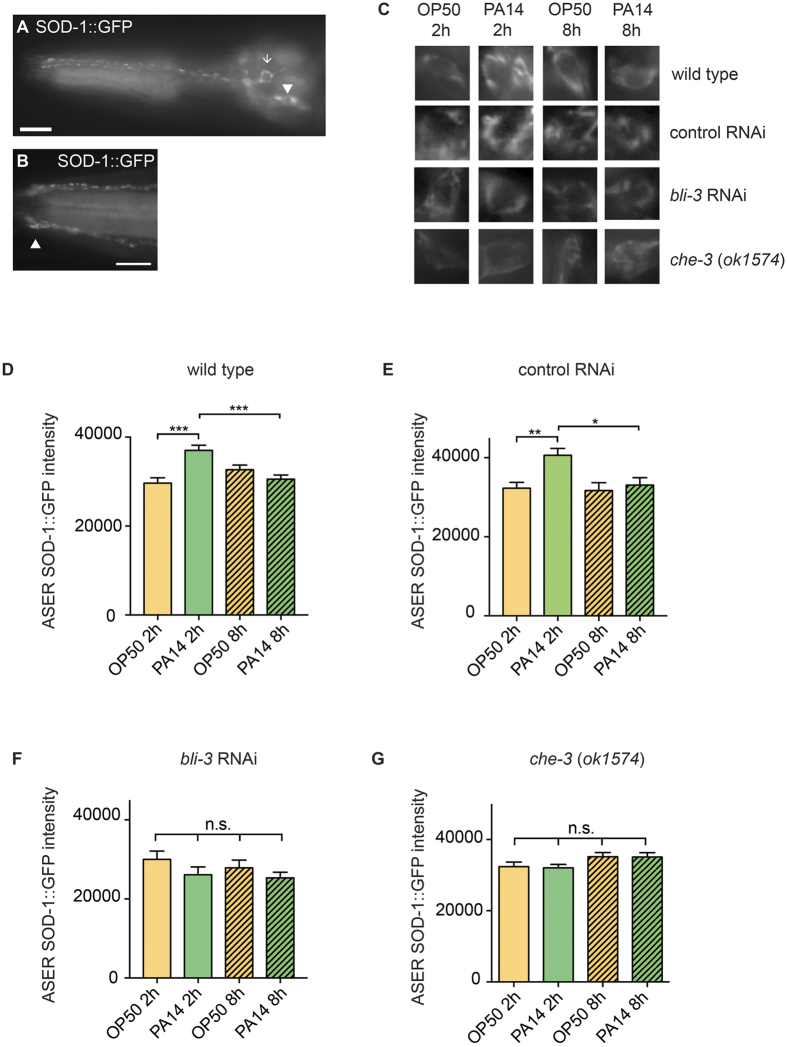
SOD-1 is temporarily induced by *P. aeruginosa* in the ASER. (**A**) Fluorescence micrographs of *bosIs2* [*sod*-*1p*::*sod*-*1* cDNA::GFP] strain. The transgenic worms were grown on *E. coli* OP50 prior to analysis. Triangle represents ASER. Arrow represents ADL. SOD-1::GFP is localized as distinct puncta along the dendrite of the ASER neuron. Scale bar indicates 10 μm. (**B**) Triangle indicates the ASER cilium. The transgenic worms were grown on *E. coli* OP50 prior to analysis. Scale bar indicates 5 μm. (**C**) Fluorescence micrographs of the ASER neuron cell body. Wild type: *sod*-*1(tm776*); *bosIs2*. Control RNAi: *sod*-*1(tm776*); *bosIs2* fed with bacteria that contain RNAi control vector. *bli*-*3* RNAi: *sod*-*1(tm776*); *bosIs2* fed with bacteria that contain *bli*-*3* RNAi. *che*-*3(ok1574*) represents *che*-*3(ok1574*); *sod*-*1(tm776*); *bosIs2*. (**D**–**G**) Average fluorescence intensity of SOD-1::GFP in the ASER neuron. ***Represents *p* < 0.001, **represents *p* < 0.01, *represents *p* < 0.05, n.s. not significant, as determined by one-way ANOVA, followed by Tukey’s multiple comparison test. *N* = 20–25. Error bars represent standard error of the mean.

SOD-1 alleviates pathogen-induced oxidative stress. Thus, we sought to determine if SOD-1 is induced by *P. aeruginosa*. We first generated an integrated *sod*-*1p*::*sod*-*1* cDNA::GFP line. We then examined the SOD-1::GFP expression pattern under normal growth conditions. We found that SOD-1::GFP fusion protein is localized as distinct puncta along the dendrite, at the sensory cilium and in the cell body of the ASER neuron (Fig. 4A). We also observed weak SOD-1::GFP signals in the intestine (Supplementary Figure 2).

Further, we transferred the SOD-1::GFP strain to a lawn of *E. coli* OP50 or to a lawn of *P. aeruginosa* PA14 and examined if SOD-1::GFP is induced. The activation of *gcs*-*1* and *gst*-*4* reporters indicates a marked increase of ROS in the intestine after 2 hour *P. aeruginosa* exposure (Fig. 3). However, we found no elevation of SOD-1::GFP signals in the intestine after 2 hour. Instead, intestinal SOD-1 expression was elevated by *P. aeruginosa* at a later time point (Supplementary Figure 2).

Because SOD-1 functions in the gustatory neuron ASER to modulate behavioral response to pathogens, we sought to examine if SOD-1 is induced in the ASER by *P. aeruginosa*. After 2 hour *P. aeruginosa* exposure, we found a 23% increase of SOD-1::GFP intensity in the ASER. However, the increase of SOD-1::GFP was short-lived. The elevated ASER SOD-1::GFP signals diminished and returned to the baseline level by 8 hour (Fig. 4C and D). In addition, we found that the reduction of SOD-1 in the ASER neuron correlates to the initiation of pathogen avoidance behavior (Fig. 1B). Wild type *C. elegans* remained on the *P. aeruginosa* lawn and started to vacate the lawn after 7 hour. In contrast, *sod*-*1(tm776*) mutant started to vacate away the *P. aeruginosa* lawn shortly after the mutant reached the source of *P. aeruginosa* (Fig. 1B and Supplementary Figure 3). Together, our data suggest that SOD-1 acts as a reactive sensor of *P. aeruginosa*. The elevation of SOD-1 in gustatory neuron ASER suppresses the aversive response to *P. aeruginosa*.

### Reactive oxygen species and sensory modality contribute to SOD-1 induction in the ASER neuron

When responding to microbial challenges, NADPH oxidase promotes the generation of ROS^6^,^11^. BLI-3 is the *C. elegans* NADPH oxidase homolog and is present predominantly in the hypodermis and intestine^50^. BLI-3 initiates pathogen-induced oxidative stress response in the intestine^4^,^8^. To investigate if pathogen-induced ROS activate SOD-1 expression, we raised SOD-1::GFP animals with bacteria that either contain RNAi control vector or *bli*-*3* RNAi. We then transferred the RNAi treated animals to a lawn of *E. coli* OP50 or to a lawn of *P. aeruginosa* PA14 and measured the fluorescence signals of SOD1::GFP at 2 hour and 8 hour. In control RNAi, we found *P. aeruginosa* elevates SOD-1::GFP expression in the ASER neuron at 2 hour. The ASER-specific SOD-1::GFP signals returned to the baseline level by 8 hour (Fig. 4C and E). These results are similar to what we observed in wild type (Fig. 4C and D). In contrast, *bli*-*3* RNAi blocked the induction of SOD-1::GFP by *P. aeruginosa* (Fig. 4C and F). Because BLI-3 activates ROS in the intestine^4^,^8^ and neuron cells are refractory to feeding RNAi treatment^51^, we conclude that ROS activate ASER-specific SOD-1 expression via a non cell-autonomous mechanism.

SOD-1::GFP is localized as distinct puncta along the dendrite and at the sensory cilium of the ASER neuron (Fig. 4A and B). We reasoned that sensory cues from external *P. aeruginosa* may induce SOD-1 expression. To test this hypothesis, we introduced SOD-1::GFP into the *che*-*3(ok1574*) deletion background. *che*-*3* encodes a dynein heavy chain motor protein. Mutation in *che*-*3* elicits chemosensory defects due to the disruption of ciliary structures in amphid sensory neurons^52^,^53^. As indicated by SOD-1::GFP, we found that *che*-*3* mutant elicits abnormal (e.g. looping and additional branching) ciliary structure of ASER neuron (Supplementary Figure 4). We further exposed *che*-*3 (ok1574*); SOD-1::GFP animals to *P. aeruginosa*. In contrast to wild type (Fig. 4C and D), we did not observe elevation of SOD-1::GFP in ASER of the *che*-*3* mutant (Fig. 4C and G). This suggests that external *P. aeruginosa* elevates SOD-1 expression in the ASER neuron. A recent report reveals that metabolites of *P. aeruginosa* modulate *C. elegans* pathogen avoidance response via activation of *daf*-*7* in ASJ sensory neuron^29^. It remains to be determined if bacterial metabolites or bacterial toxins serve as the external cues to elevate SOD-1 expression in the ASER gustatory neuron.

### Antagonistic sensory modality functions in parallel to activate aversive response to pathogens

**Figure 5:**
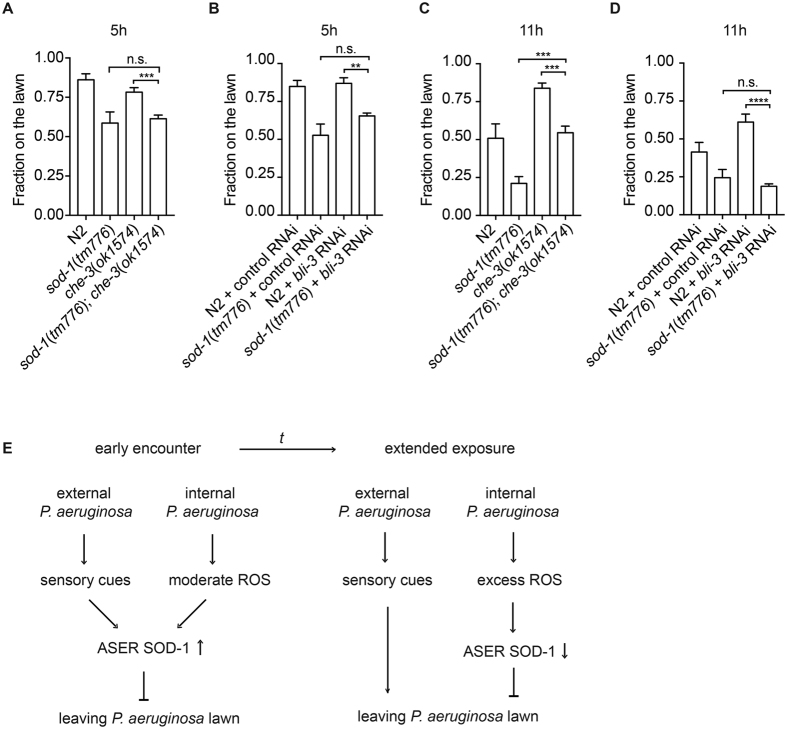
SOD-1 integrates signals from internal ROS and external stimuli to repress *P. aeruginosa* avoidance behavior. (**A**–**D**) *P. aeruginosa* lawn occupancy of *C. elegans* strains assayed at 5 h and 11 h. ****Represents *p* < 0.0001, ***represents *p* < 0.001, **represents *p* < 0.01, n.s. not significant, as determined by one-way ANOVA, followed by Tukey’s multiple comparison test. *N* = 9–15. Error bars represent standard error of the mean. (**E**) When *C. elegans* first encounters *P. aeruginosa*, internal ROS and external sensory stimuli elevate SOD-1 expression in ASER. In turn, the induction of SOD-1 in ASER blocks behavioral avoidance of *P. aeruginosa*. After prolonged exposure to *P. aeruginosa*, SOD-1 expression in ASER returns to basal level. The inhibition of *P. aeruginosa* avoidance behavior is possibly lifted via a mechanism that is in parallel of the *sod*-*1* dependent response.

To examine if sensory modalities and ROS play a role in modulating *C elegans* aversive behavior to pathogens, we investigated the pathogen avoidance phenotypes of *che*-*3* mutant animals and *bli*-*3* RNAi animals. We found that *che*-*3* mutant animals and *bli*-*3* RNAi animals elicit delayed aversive responses to *P. aeruginosa* (Fig. 5). We further tested if the *sod*-*1* mutation can reverse the delayed behavioral phenotype of *che*-*3(ok1574*) and *bli*-*3 RNAi*. At 5 hour, we found that *sod*-*1(tm776*); *che*-*3(ok1574*) and *sod*-*1(tm776*); *bli*-*3* RNAi elicit heightened pathogen avoidance phenotype similar to *sod*-*1(tm776*) (Fig. 5A and B). These results are consistent with our observations that SOD-1 is induced in the ASER via CHE-3 and BLI-3 dependent signaling (Fig. 4C,E–G). Intriguingly, at 11 hour, as *sod*-*1* mutant continued to block the behavioral response caused by *bli*-*3* RNAi, *sod*-*1(tm776*); *che*-*3(ok1574*) double mutant elicited intermediate behavioral phenotype (Fig. 5C and D). Since mutation of *che*-*3* disrupts many sensory neurons, the intermediate phenotype of *sod*-*1*; *che*-*3* double mutant may be contributed by the inactivation of additional sensory modality^28^,^29^,^30^. Our results suggest that ROS mediate *P. aeruginosa* avoidance in a *sod*-*1* dependent manner. On the other hand, external sensory stimuli initially elevate SOD-1 expression in the gustatory neuron ASER. After prolonged *P. aeruginosa* exposure, antagonistic sensory modality promotes pathogen avoidance behavior and allows *C. elegans* to exit the lawn of *P. aeruginosa* (Fig. 5E).

Together, our results demonstrate that SOD-1 functions in the gustatory neuron ASER to mediate *C. elegans* pathogen avoidance behavior. SOD-1 is localized along the dendrite, at the cilium and in the cell body of the ASER neuron. SOD-1 is induced by *P. aeruginosa*. Elevation of SOD-1 inhibits the avoidance response towards *P. aeruginosa*. After prolonged *P. aeruginosa* exposure, reduction of SOD-1 in the ASER allows *C. elegan*s to vacate the *P. aeruginosa* lawn. We also found that ROS modulate *P. aeruginosa* avoidance in a *sod*-*1* dependent and non cell-autonomous manner. The activation of antagonistic signaling modalities in the sensory system may contribute to behavior plasticity and play a modulatory role in regulating the timing of pathogen avoidance.

## Discussion

Interactions between the brain, the gut and enteric microbes are vital for maintaining physiological homeostasis^15^,^54^. Microbiota in the intestine affect obesity, type 2 diabetes and inflammatory bowel disease manifestation^55^,^56^,^57^. Alterations in brain-gut interaction are also associated with stress response and behavior^58^,^59^,^60^,^61^. Recent advancement in human microbiome research suggests that the composition of enteric microbes and the signaling triggered by enteric microbes from gut to the central nervous system is important to our health^16^,^57^. Therefore, it has been coined that the gut and enteric microbes are our “second brain”^62^. Currently, the lack of whole-animal models has impeded the progress to understand how gut microbes modulate brain physiology.

*C. elegans* is a well-established organism to study neurobiology^63^,^64^,^65^,^66^,^67^ and host-microbe interactions^28^,^68^,^69^,^70^,^71^,^72^. Compared to the mammalian brain, which contains billions of neurons, there are only 302 neurons in *C. elegans*. Most importantly, the neural circuitry of *C. elegans* has been extensively characterized to the resolution of a single neuron^73^,^74^. *C. elegans* can grow axenically or can feed on bacteria. Prior to entering the intestine of *C. elegans*, bacteria are normally broken down in the pharynx. However, many human opportunistic pathogens, including *P. aeruginosa*, colonize the worm intestine^17^,^22^,^75^,^76^. When *C. elegans* first encounters *P. aeruginosa, C. elegans* will remain inside the bacterial lawn and feed on *P. aeruginosa*^28^. Ingestion allows the accumulation of *P. aeruginosa* in the intestine^17^ and triggers ROS production^4^,^5^.

We found after 2 hour exposure to *P. aeruginosa*, the fluorescence signals of oxidative stress reporters *gcs*-*1p*::GFP and *gst*-*4p*::GFP are elevated in the intestine (Fig. 3). These results support that *P. aeruginosa* triggers ROS generation at the mucosal membrane in the intestine. In addition, we abolished the elevation of SOD-1 in the ASER neuron by knocking down *bli*-*3* outside of the nervous system (Fig. 4C and F). This suggests that ROS induce SOD-1 expression in the ASER via a non cell-autonomous mechanism. ROS may act directly or may activate long-range signaling molecules at the brain-gut axis to modulate the pathogen avoidance response. Given the small size of *C. elegans*, many chemicals from the environment activate stress response in distal tissue via diffusion^39^,^77^,^78^. Excess intestinal ROS may cause elevation of systemic ROS and subsequently induce SOD-1 in the nervous system. In addition, the intestine-derived neuropeptides are potential long-range signaling molecules to mediate SOD-1 expression in the ASER. Further study is needed to confirm if neuroendocrine signaling plays a role in modulating SOD-1 levels in the nervous system.

Our results reveal that external *P. aeruginosa* mediates SOD-1 induction in the ASER neuron (Fig. 4C and G). the dendritic ending of ASER neuron is directly exposed to the environment. Therefore, ASER neuron may serve to detect bacterial toxins or metabolites secreted by *P. aeruginosa*. It has been postulated that the *C. elegans* behavioral response to pathogens is controlled by external sensory modalities and intracellular stress signaling^79^. Consistent with this hypothesis, our findings suggest that external sensory stimuli modulate ROS-induced *sod*-*1* dependent pathogen avoidance behavior in *C. elegans*.

A recent study in budding yeast highlights a role of SOD-1 in maintaining respiratory homeostasis and glucose metabolism^38^. SOD-1 suppresses the degradation of casein kinase 1, a signaling molecule that represses respiratory metabolism. Our results suggest that in a multicellular organism, SOD-1 is induced in a gustatory neuron by ROS (Fig. 4). SOD-1 represses *C. elegans* behavioral response to *P. aeruginosa*, which in turn triggers generation of more ROS in the intestine (Fig. 1 and Supplementary Figure 2). After prolonged *P. aeruginosa* exposure, the reduction of neuronal SOD-1 allows *C. elegan*s to vacate the *P. aeruginosa* lawn (Figs 1 and 4). By reducing ROS, we were unable to ameliorate the heightened pathogen avoidance phenotype of *sod*-*1* deletion (Fig. 5). Our data indicate that SOD-1 plays a key role in the nervous system to mediate pathogen avoidance behavior in response to the internal state of redox homeostasis.

When food sources are scarce, *sod*-*1* dependent behavior may provide survival benefits by delaying the aversive response thus allowing *C. elegans* to take advantage of food that is contaminated with harmful microbes. Our previous findings reveal that naturally occurring polymorphisms in *npr*-*1* (a neuropeptide Y receptor homolog) and *hecw*-*1* (a neuron specific E3 ligase) can lead to changes in behavior that may facilitate the adaptation of *C. elegans* to microbes in nature^28^. In a recent survey of microbiome in *C. elegans* natural habitats, it reveals that 22% of the bacterial species are detrimental to *C. elegans* growth. Wild *P. aeruginosa* strains are among the identified detrimental species, and these wild *P. aeruginosa* strains activate *C. elegans* pathogen response^2^. Given that 78% of the natural bacteria species are possible food sources, the ability to continue feeding when small quantities of toxic bacteria are present is beneficial to survival. We hypothesized that the activation of SOD-1 in the gustatory neuron may provide an evolutionarily conserved strategy by allowing animals to take advantage of non-ideal food.

SOD-1 is an enzyme that converts superoxide into less toxic hydrogen peroxide and oxygen. Investigation of mammalian SOD-1 reveals that SOD-1 is a stable enzyme with a slow turnover rate in the central nervous system. The slow turnover rate of wild type SOD-1 may explain the susceptibility of the nervous system in ALS neurodegenerative proteinopathies^80^. Although SOD-1 expression is not elevated in the intestine during the early stage of pathogen exposure, we found SOD-1 is elevated in the intestine at 8 hour (Supplementary Figure 2). This suggests that during prolonged exposure to pathogens, ROS are generated continuously. We speculate that excess systemic ROS may activate the turnover of SOD-1 in the nervous system. Our data demonstrate a link between SOD-1 protein level and animal behaviors. Given the high degree of genomic conservation, our results may shed new light on the function of SOD-1 in humans and lead to a more thorough understanding of ALS pathology.

## Materials and Methods

### Strains

*C. elegans* strains were maintained at 20 °C using standard methods^81^. Strains were maintained at 20 °C then shifted to 22.5 °C for *P. aeruginosa* PA14 lawn avoidance assays. The *sod*-*1(tm776*) mutant strain was derived from GA187 strain (obtained from the Caenorhabditis Genetics Center) by backcrossing it six times to N2. Transgenic strains were isolated by microinjecting various plasmids, (typically at 50–100 μg/ml) together with one of the following coinjection markers, *rol*-*6*dm, *myo*-*2p*::mstrawberry, *unc*-*122p*::GFP, and *unc*-*122p*::mcherry in wild type or mutant animals. UV integration of extrachromosomal array was performed following the protocol originated from S. Mitani. The integrated lines were then backcrossed six times to N2 prior to the analysis.

### *P. aeruginosa* PA14 avoidance assay

A 100 mL solution of LB was inoculated with a single colony of *P. aeruginosa* PA14 and grown overnight without shaking at 37 °C until O.D. reaches 0.2–0.3. 30 μl of this culture was used to seed the center of the 100-mm NGM plate. Seeded plates were incubated for 24 h at room temperature (22.5 °C) prior to the experiment. Approximately 30 larval stage 4 (L4) animals were transferred onto plates containing the *P. aeruginosa* PA14 lawn at 22.5 °C, and lawn occupancy was measured at the indicated times. Three plates of each genotype were performed in each experiment and all experiments were performed at least three times. Upon being transferred to the *P. aeruginosa* PA14-containing plates, animals explored the plate for about 10–15 minutes until they found the bacterial lawn and then remained in the lawn. Subsequently, lawn occupancy was measured over time as the lawn avoidance behavior is observed^28^.

### Molecular cloning

The genomic region of *sod*-*1* was amplified by PCR using primers 5′-GAACACCAAACCGGACTGACCAAGT-3′ and 5′-GTTTATGACGCAAAGCGTACGGACAATCTC-3′. The 2.9 kb genomic fragment was cloned into a Topo^®^ (Invitrogen) vector. The *sod*-*1* promoter region was amplified by using a 5′ primer containing 5′-GAACACCAAACCGGACTGACCAAGT-3′ and 3′ primer containing 5′-CAAAGTTGTAGATTCAGTATTTTAGATCGGTG-3′. The 1223 bp fragment was subsequently cloned into the pPD95.75 vector (Fire Lab Vector Kit, Addgene) to generate the *sod*-*1* promoter GFP reporter. *sod*-*1* cDNA clones are gifts from Y. Kohara (yk524g1, yk593d7, yk1381e03 and yk1715f05). The *unc*-*119, ges*-*1, flp*-*6, sre*-*1, gcy*-*5* promoters were generated using primers as described previously^40^,^41^,^82^,^83^,^84^. *csp*-*1b* cDNA is a gift from D. Denning and H.R. Horvitz^46^. Detailed primer sets and methods used for cloning are available upon request.

### Microscopy

Animals were mounted in M9 with levamisole (10 mM) onto slides with a 3% agarose pad. The slides were viewed using an AxioImager Z1 fluorescence microscope (Zeiss) with 10x/0.25, 40x/0.75 and 63x/1.4 (oil) objectives. The fluorescence signals were recorded by a CCD camera in a 16 bit format without saturation. The images were captured and analyzed by ProgRes imaging software. Fluorescence intensity was measured and calculated using Image-Pro software.

### RNAi treatments

RNAi treatments were performed by feeding *C. elegans* RNAi constructs and reagents described previously^85^,^86^. HT115 *E. coli* carrying RNAi clones in the pL4440 vector were cultured overnight in LB liquid media with antibiotics. NGM plates containing 1 mM IPTG and antibiotics were seeded with the feeding RNAi bacterial culture. The plates were incubated at 20 °C for bacteria growth. Five L2 larvae were placed onto each seeded NGM plate, and the progeny of these animals were scored for blister phenotype prior to *P. aeruginosa* PA14 avoidance assay to confirm the effectiveness of the RNAi treatment.

### Statistical analysis

Statistical analysis was performed using GraphPad Prism software.

## Additional Information

**How to cite this article:** Horspool, A. M. and Chang, H. C. Superoxide dismutase SOD-1 modulates *C. elegans* pathogen avoidance behavior. *Sci. Rep.* **7**, 45128; doi: 10.1038/srep45128 (2017).

**Publisher's note:** Springer Nature remains neutral with regard to jurisdictional claims in published maps and institutional affiliations.

## Supplementary Material

Supplementary Figures
