# Supplementary material for: Superoxide dismutase SOD-1 modulates C. elegans pathogen avoidance behavior
Source: Sci Rep. 2017 Mar 21;7:45128. doi: 10.1038/srep45128 (PMC5359715; doi:10.1038/srep45128)
Supplement: Supplementary Figures [file srep45128-s1.pdf]

Superoxide dismutase SOD-1 modulates *C. elegans* pathogen avoidance behavior

Horspool AM. and Chang HC.

Sup. Fig. 1

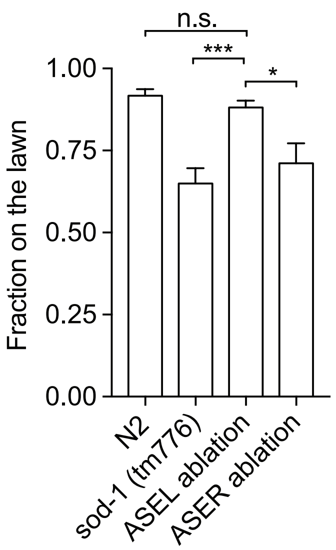

Supplementary Figure 1. Ablation of ASEL neuron does not trigger heightened behavioral response to *P. aeruginosa.*

P. aeruginosa lawn occupancy of *C. elegans* strains assayed at t = 5h. *** represents $p<0.001$,* represents $p<0.05$, n.s. not significant, as determined by one-way ANOVA, followed by Tukey's multiple comparison test. *N*=9-12. Error bars represent standard error of the mean.

Superoxide dismutase SOD-1 modulates *C. elegans* pathogen avoidance behavior

Horspool AM. and Chang HC.

Sup. Fig. 2

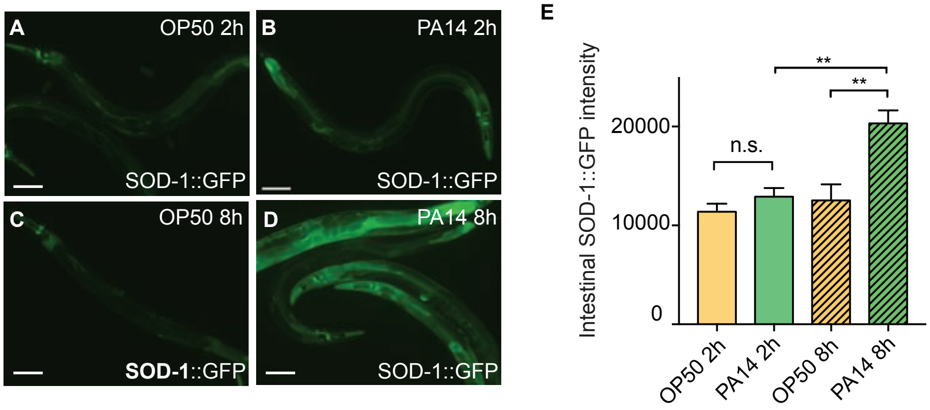

Supplementary Figure 2. SOD-1 is induced in the intestine after extended exposure to *P. aeruginosa.*

(A-D) Fluorescence micrographs of intestinal SOD-1::GFP. *sod-1*(*tm776*); *bosIs2* [*sod-1p*::*sod-1* cDNA::GFP] is exposed to *E. coli* OP50 or to *P. aeruginosa* PA14. Scale bar indicates 50 μm.

(E) Average fluorescence intensity of SOD-1::GFP in the intestine. ** represents p<0.01, n.s. not significant, as determined by one-way ANOVA, followed by Tukey's multiple comparison test. *N*=18-20. Error bars represent s.e.m.

Superoxide dismutase SOD-1 modulates *C. elegans* pathogen avoidance behavior

Horspool AM. and Chang HC.

Sup. Fig. 3

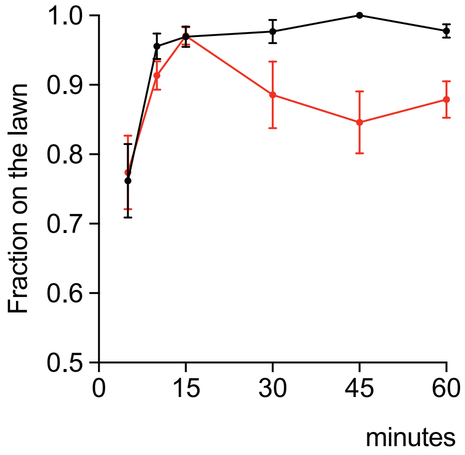

Supplementary Figure 3. *P. aeruginosa* lawn occupancy of N2 and *sod-1*(*tm776*)

Time course of the *P. aeruginosa* lawn occupancy. Black indicates wild-type N2 strain; red indicates *sod-1*(*tm776*) mutant strain. *N*=12. Error bars represent standard error of the mean.

Superoxide dismutase SOD-1 modulates *C. elegans* pathogen avoidance behavior

Horspool AM. and Chang HC.

Sup. Fig. 4

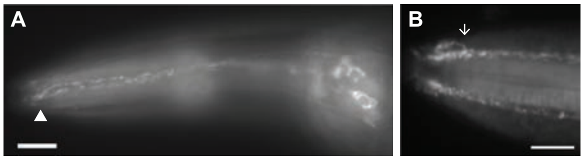

Supplementary Figure 4. *che-3*(*ok1574*) mutant elicits abnormal ASER cilium structure.

(A-B) Fluorescence micrographs of *bosIs2* [*sod-1p*::*sod-1* cDNA::GFP]; *che-3*(*ok1574*). Looping (triangle) and branching (arrow) of the ASER cilium is shown. Scale bar indicates 10 μm in (A) and 5 μm in (B).
